# Efficacy of IVIG therapy for patients with sepsis: a systematic review and meta-analysis

**DOI:** 10.1186/s12967-023-04592-8

**Published:** 2023-10-28

**Authors:** Bo Pan, Pan Sun, Renjun Pei, Fangzhao Lin, Haijun Cao

**Affiliations:** Institute of Blood Transfusion, Chinese Academy of Medical Sciences and Peking Union Medical College, Chengdu, 610052 China

**Keywords:** IVIG, Sepsis, Age-difference, Economic regions, Meta-analysis

## Abstract

**Background:**

Sepsis is an overwhelming reaction to infection that comes with high morbidity and mortality. It requires urgent interventions in order to improve outcomes. Intravenous immunoglobulins (IVIG) are considered as potential therapy in sepsis patients. Results of trials on IVIG as adjunctive therapy for sepsis have been conflicting due to the variability in population characteristics, country geography and drug dosage form in different studies.

**Methods:**

A systematic article search was performed for eligible studies published up to January, 31, 2023, through the PubMed, Embase, Cochrane Library and Chinese National Knowledge Infrastructure database. The included articles were screened by using rigorous inclusion and exclusion criteria. Subgroup analyses were conducted according to different IVIG types, ages and economic regions. All analyses were conducted using Review Manager 5.4. Quality of studies and risk of bias were evaluated.

**Results:**

In total, 31 randomized controlled trials were included with a sample size of 6,276 participants. IVIG could reduce the mortality (RR 0.86, 95% CI: 0.77–0.95, *p* = 0.005), the hospital stay (MD − 4.46, 95% CI: − 6.35 to − 2.57, *p* = 0.00001), and the APACHE II scores (MD − 1.65, 95% CI: − 2.89 to − 0.63, *p* = 0.001). Additionally, the results showed that IgM-enriched IVIG was effective in treating sepsis (RR 0.55, 95% CI: 0.40 − 0.76; *p* = 0.0003), while standard IVIG failed to be effective (RR 0.91, 95% CI: 0.81–1.02,* p* = 0.10). And the effect of IVIG in reducing neonatal mortality was inconclusive (RR 0.93, 95% CI: 0.81–1.05, *p* = 0.24), but it played a large role in reducing sepsis mortality in adults (RR 0.70, 95% CI: 0.57–0.86, *p* = 0.0006). Besides, from the subgroup of different economic regions, it indicated that IVIG was effective for sepsis in high-income (RR 0.89, 95% CI: 0.79–0.99,* p* = 0.03) and middle-income countries (RR 0.49, 95% CI: 0.28–0.84,* p* = 0.01), while no benefit was demonstrated in low-income countries (RR 0.56, 95% CI: 0.27–1.14,* p* = 0.11).

**Conclusions:**

There is sufficient evidence to support that IVIG reduces sepsis mortality. IgM-enriched IVIG is effective in both adult and neonatal sepsis, while standard IVIG is only effective in adult sepsis. IVIG for sepsis has shown efficacy in high- and middle-income countries, but is still debatable in low-income countries. More RCTs are needed in the future to confirm the true clinical potential of IVIG for sepsis in low-income countries.

**Supplementary Information:**

The online version contains supplementary material available at 10.1186/s12967-023-04592-8.

## Introduction

Sepsis is a syndrome of life-threatening organ dysfunction caused by a dysregulated host response to infection [1]. It affects more than 31.5 million people worldwide each year, with potentially 5.3 million deaths annually [2]. When sepsis is combined with severe circulatory dysfunction, cellular and metabolic disturbances, it can develop into severe sepsis and septic shock [3, 4]. Despite the development of new and effective antibiotics, the in-hospital mortality from sepsis remains high [5, 6]. It showed no significant effect of antibiotic timing or early antibiotic use on mortality benefit from sepsis [7]. In addition, drugs targeting TNF-α, IL-1β, or toll-like receptors have not achieved satisfactory clinical efficacy in improving the survival rate of patients with sepsis [8]. No effective specific anti-sepsis treatments exist [9], therefore further studies on interventions for sepsis is urgent.

Intravenous immunoglobulin (IVIG), containing the full range of antibody spectrum, is derived from the plasma of thousands of healthy donors [10]. It contains antibodies against pathogenic microorganisms to which the body is susceptible [10]. And it is used in clinical practice to treat inflammatory diseases such as severe infections and neonatal sepsis. Lipid A in bacterial endotoxins is considered to be the main toxic component in the systemic inflammatory response to sepsis [11]. The use of antibodies against different components of endotoxins as adjunctive therapy for sepsis has been the target [12, 13]. IVIG has been reported to treat sepsis, but its clinical efficacy remains controversial [12]. It was indicated that treatment with IVIG may be associated with lower sepsis mortality but the evidence base shows a large degree of heterogeneity between individual studies [14]. The large degree of heterogeneity in treatment effects between studies could be explained by a measure of study quality and IVIG dosing regimen [15]. Similarly, in 2016 surviving sepsis campaign suggested against IVIG use in sepsis due to the low certainty of evidence and the significant heterogeneity [16]. Nevertheless, it still encouraged conduct of large multicenter studies to further evaluate the effectiveness of other IVIG in patients with sepsis. Thus updating the included literature and exploring sources of heterogeneity to provide the latest evidence on the efficacy of IVIG for sepsis is warranted. Just as the revised pyramid of evidence considers systematic reviews as lenses for viewing (applying) evidence [17], the real-time and high-quality systematic reviews of RCTs can provide complementary and information for the subsequent development of guidelines on sepsis.

To date, the factors that influence the efficacy of IVIG for sepsis have not been thoroughly explored. It was reported that different types of IVIG are key to the efficacy of sepsis. The pentameric structure of IgM improves the activation of the complement system compared to IgG in standard IVIG [18]. Thus, IgM-enriched IVIG has higher antimicrobial activity and opsonisation. Besides, age is another important factor in the efficacy of treating sepsis. Neonatal sepsis presents with nonspecific signs and symptoms compared to adults [19]. And current diagnostic methods rely on conventional culture methods for neonatal sepsis, which is time-consuming, and may delay critical therapeutic decisions. Of importance, precise estimates of sepsis burden varied by setting. Differing estimates of disease burden have been reported from high-income countries compared with reports from low-income and middle-income countries [20]. In recent years, clinical trials have provided new evidence on the efficacy of IVIG for sepsis. Therefore, it is important to emphasise that differences in mortality from sepsis involve the variability in different IVIG types, ages and economic regions in different studies [15]. Given that difference in the efficacy of IVIG for sepsis remains incompletely clear in relation to these factors, exploring the underlying connections is warranted.

This study conducted a meta-analysis of the efficacy of IVIG in the treatment of sepsis based on patients ages, IVIG types and economic regions, providing a basis for the application of IVIG in clinical treatment.

## Methods

This study was designed and conducted according to the Preferred Reporting Items for Systematic Reviews and Meta-analyses (PRISMA) reporting guideline [21]. The present protocol has been registered within the PROSPERO database (registration number CRD42023395749).

### Design and search strategy

The search included articles in English language published in the PubMed, Embase, Cochrane library, Chinese National Knowledge Infrastructure database through January, 31, 2023. The search was conducted using the following keywords: Intravenous Immunoglobulin or IVIG or Intravenous Antibodies and Sepsis or Septicemia or Pyemia or Septic Shock and randomized controlled trial or controlled clinical trial or placebo or groups. The detailed retrieval strategy can be found in the Additional file 1.

### Criteria for inclusion and exclusion

Inclusion criteria for the systematic review were (1) randomized controlled study of sepsis; (2) all subjects were diagnosed with sepsis; (3) experimental group was not given intervention other than IVIG administration under the guarantee of basic medical care. Studies were excluded if (1) study design was not an RCT; (2) study reported insufficient details to derive the study outcomes; (3) study had other interventions; (4) the full text of the study was not available in the databases; (5) study was on an animal model.

### Study outcomes

We assessed the primary outcomes of this study is an efficacy measure, all-cause mortality at the end of the follow-up period. The secondary outcomes including the length of hospital stay among survivors and APACHE II, were assessed for efficacy.

### Data extraction

Two investigator (PB, LFZ) performed the literature search and screening, and 2 investigators (SP, RJP) independently performed data extraction. Discrepancies were resolved through discussion between investigators. The extracted data items include (1) study design, country, year of publication; (2) participant characteristics, including age, size, source, income level; (3) details of the intervention, treatment duration. An additional file shows this in more detail (Additional file 2). The location of authors institute affiliations for all papers was classified into high-, middle-, or low-income countries based on the World Bank Classification system [22].

### Risk of bias

**Fig. 1:**
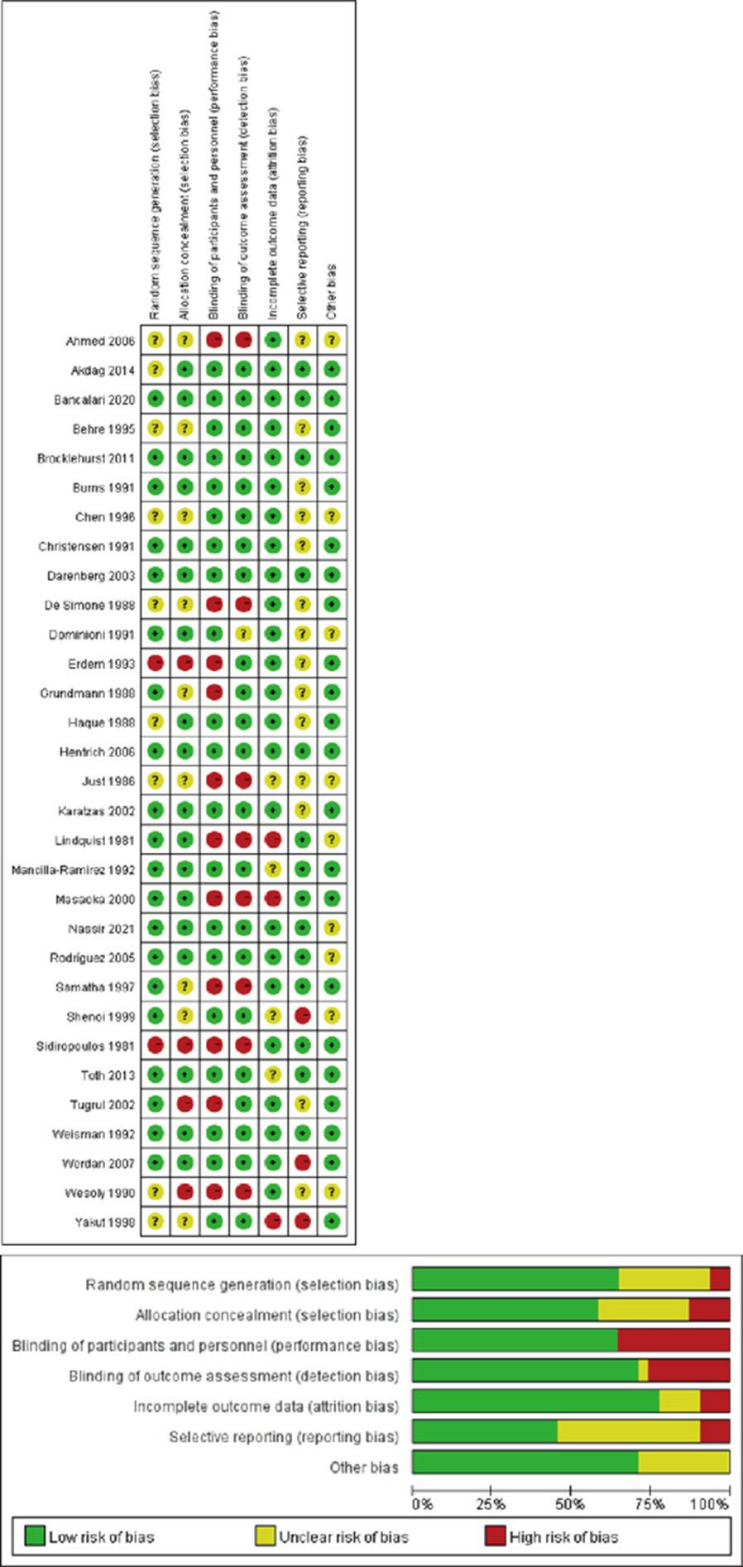
Assessment for risk of bias in included studies

We scored the studies that met inclusion criteria according to the Cochrane risk of bias tool [15], which evaluated the random sequence generation, allocation concealment, blinding of participants, personal and outcome assessment, incomplete outcome data, selective outcome reporting, and other biases (Fig. 1). The included RCTs were classified as low risk (L), high risk (H) or unclear risk (U) in the above items.

## Results

**Fig. 2:**
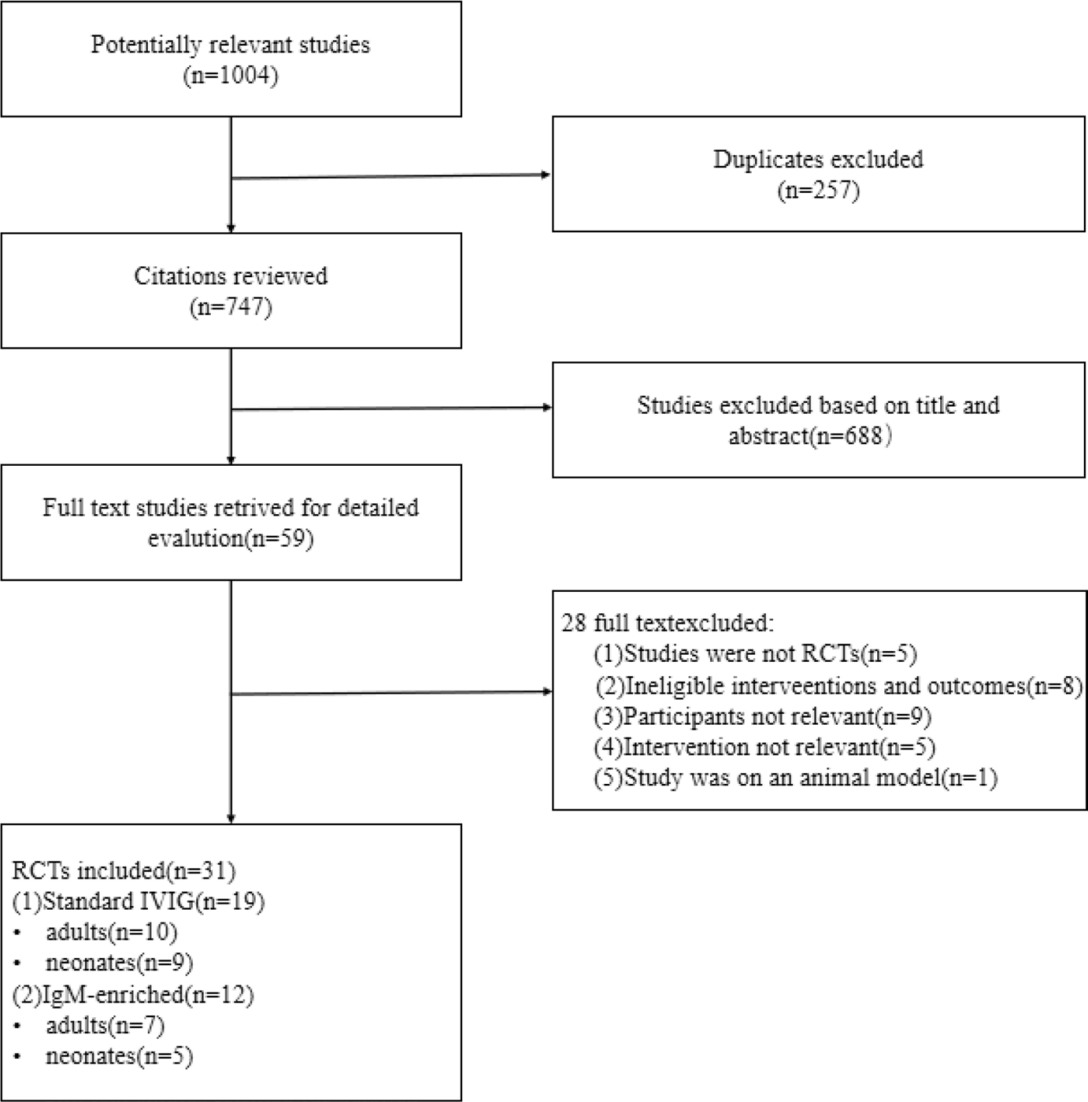
Study flow diagram

A total of 1004 references were identified from the databases (Fig. 2). After excluding duplications and screening of titles and abstracts, the full papers of 59 studies were obtained and assessed for eligibility. According to the inclusion criteria, 31 studies [18, 23–52] were finally included. Of the 31 RCTs, 19 were standard IVIG and 12 were IgM-enriched IVIG. The types of IVIG products were further subdivided, including 10 standard IVIG for adults, 7 IgM-enriched IVIG for adults, 9 IVIG for newborns, and 5 IgM-enriched IVIG for newborns. Out of the trials, mortality was assessed in all 31 RCTs after randomization. Length of hospital stay was available in 7 RCTs. And 5 RCTs assessed APACHE II scores.

**Fig. 3:**
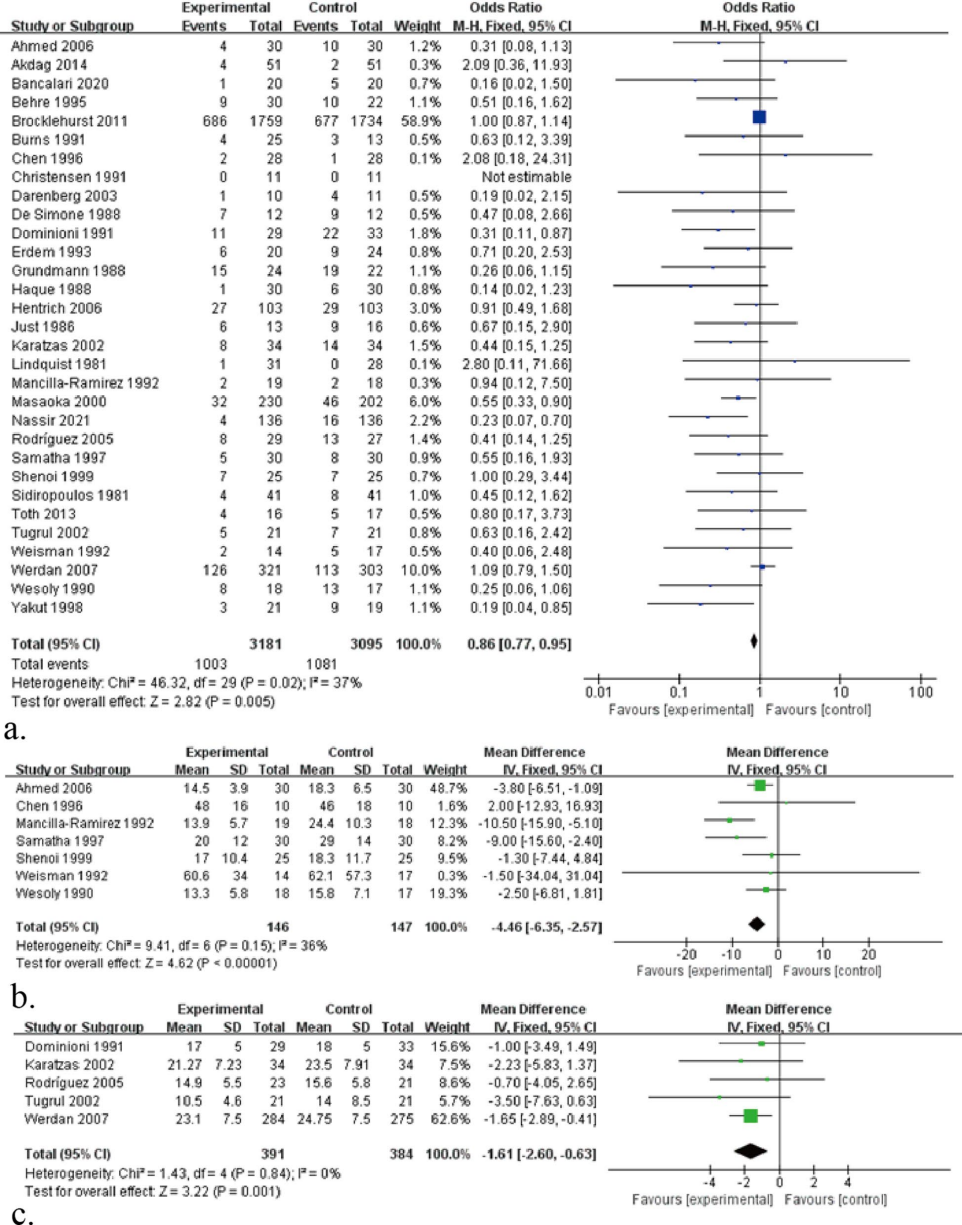
**a** Forest plots of all-cause mortality (31 RCTs, n = 6276). **b** Forest plots of the length of hospital stay (7 comparisons, n = 293). **c** Forest plots of APACHE II scores (5 RCTs, n = 775)

**Fig. 4:**
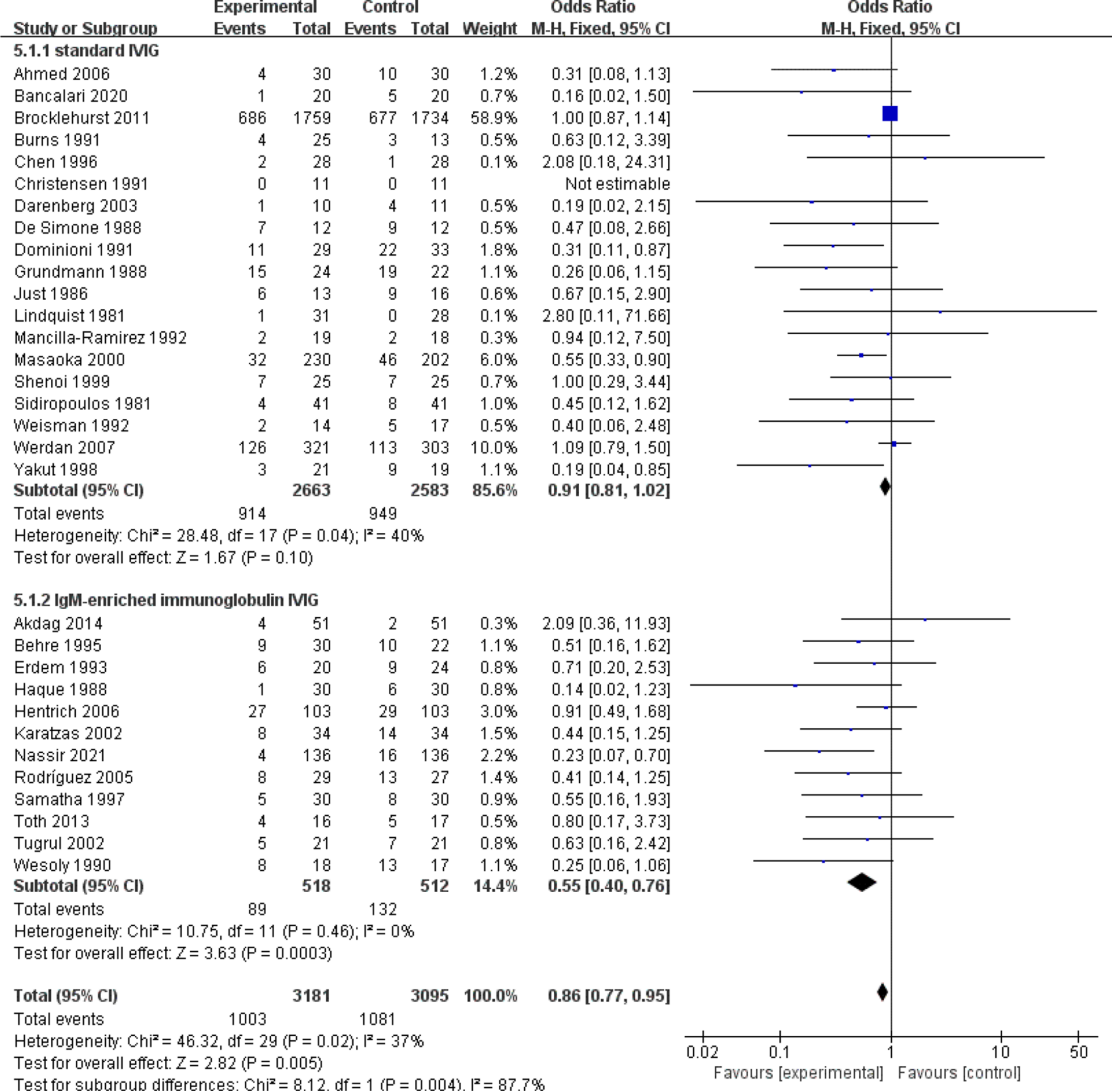
Forest plots of all-cause mortality in different IVIG types (31 RCTs, n = 6276)

**Fig. 5:**
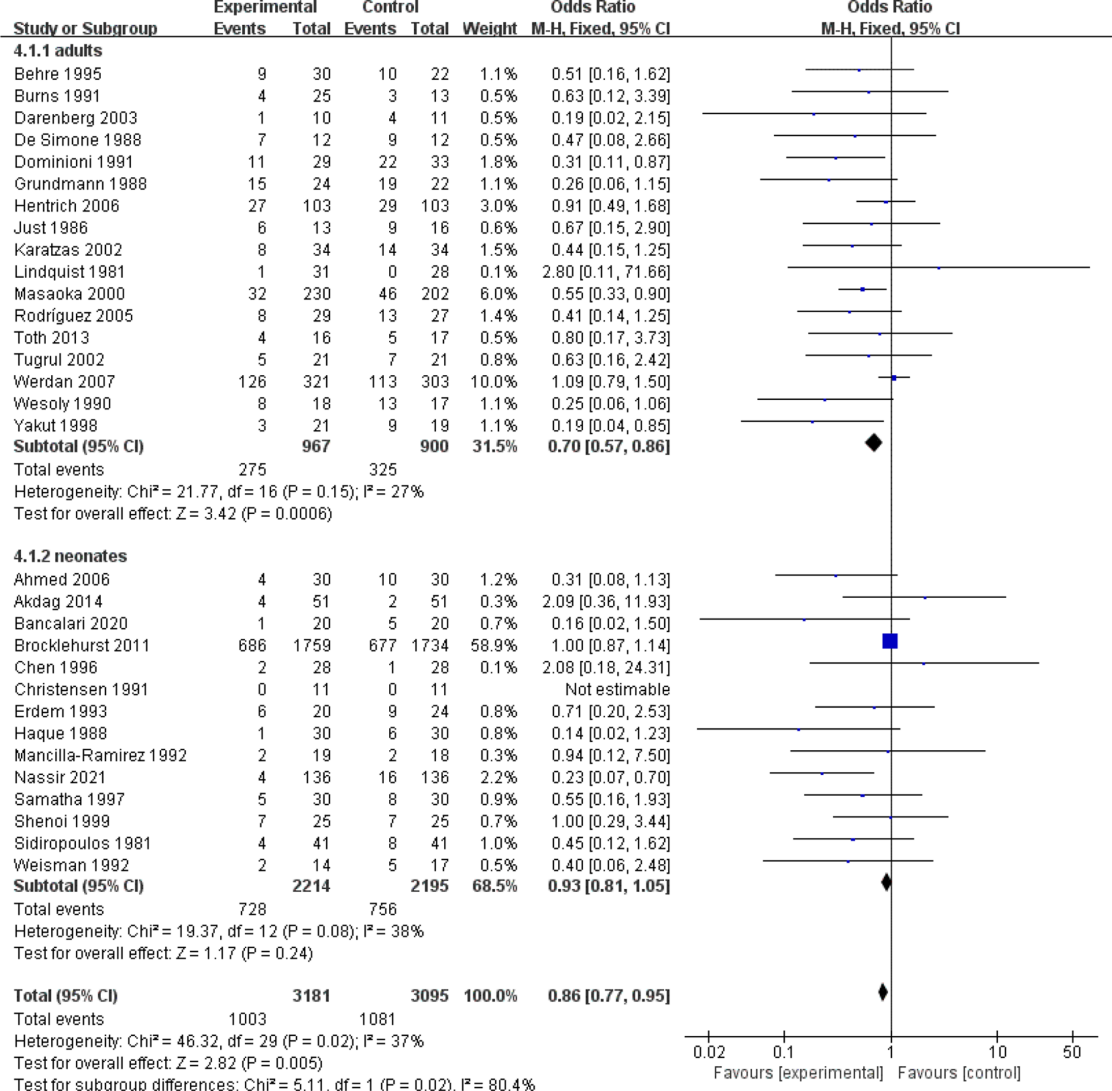
Forest plots of all-cause mortality in 2 different ages (31 RCTs, n = 6276)

**Fig. 6:**
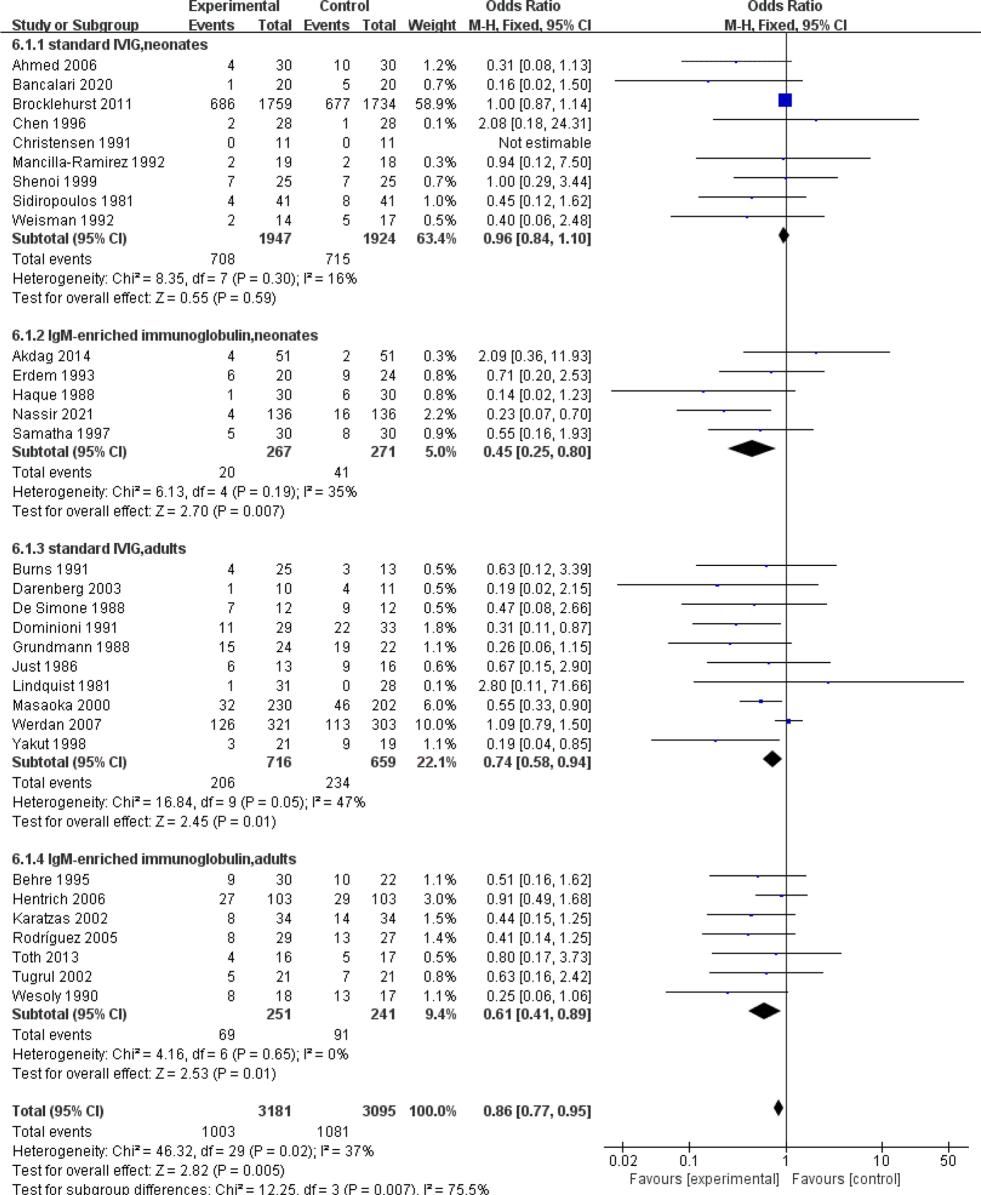
Forest plots of all-cause mortality in different IVIG types combined with 2 different ages (31 RCTs, n = 6276)

All-cause mortality in sepsis is an important indicator for the analysis of sepsis by drug therapy, so this study also focuses on all-cause mortality after IVIG treatment for sepsis. Evidence from 31 RCTs (n = 6276) of sepsis patients showed IVIG did play a great role in reducing the mortality (Fig. 3a): RR 0.86, 95% CI: 0.77–0.95; heterogeneity χ^2^ = 46.32, df = 29, *p* = 0.02, *I*^*2*^ = 37%. Besides, the results showed that IgM-rich IVIG was effective in treating sepsis (Fig. 4): RR 0.55, 95% CI: 0.40–0.76; heterogeneity χ^2^ = 10.75, df = 11, *p* = 0.46, *I*^*2*^ = 0%, while standard IVIG failed to be effective (Fig. 4): RR 0.91, 95% CI: 0.81–1.02; heterogeneity χ^2^ = 28.48, df = 17, *p* = 0.04, *I*^*2*^ = 40%. Therefore, to assess the effect of IVIG treatment for sepsis in age-differentiated groups of all-cause mortality, we performed subgroup analyses of participants by 2 different ages. It showed that the effect of IVIG in reducing neonatal mortality was inconclusive (Fig. 5): RR 0.93, 95% CI: 0.81–1.05; heterogeneity χ^2^ = 19.37, df = 12, *p* = 0.08, *I*^*2*^ = 38%, but it played a large role in reducing sepsis mortality in adults (Fig. 5): RR 0.70, 95% CI: 0.57–0.86; heterogeneity χ^2^ = 21.77, df = 16, *p* = 0.15, *I*^*2*^ = 27%. To further analyse whether different IVIG types have a positive effect on the treatment of neonatal sepsis, we grouped 14 RCTs on neonates, 9 of which were standard IVIG and 5 were IgM-enriched IVIG. The results showed that there was no correlation between the standard IVIG group and the treatment effect of neonatal sepsis (Fig. 6): RR 0.96, 95% CI: 0.84–1.10; heterogeneity χ^2^ = 8.35, df = 7, *p* = 0.30, *I*^2^ = 16%. However, the IgM-rich IVIG group showed a positive effect in the treatment of neonatal sepsis (Fig. 6): RR 0.45, 95% CI: 0.25–0.80; heterogeneity χ^2^ = 6.13, df = 4, *p* = 0.19, *I*^*2*^ = 35%.

7 RCTs with 293 patients were available for length of hospital stay in this meta-analysis. Compared with the control group, the IVIG treatment group significantly reduced the length of hospital stay in patients with sepsis (Fig. 3b): MD − 4.46, 95% CI: − 6.35 to − 2.57; heterogeneity χ^2^ = 9.41, df = 6, *p* = 0.15, *I*^*2*^ = 36%.

5 RCTs assessed APACHE II scores, an indicator of prognosis in sepsis survivors. The results showed that the IVIG treatment group significantly reduced the APACHE II score in patients with sepsis (Fig. 3c): MD − 1.65, 95% CI: − 2.89 to − 0.63; heterogeneity χ^2^ = 1.43, df = 4, *p* = 0.85, *I*^2^ = 0%.

**Fig. 7:**
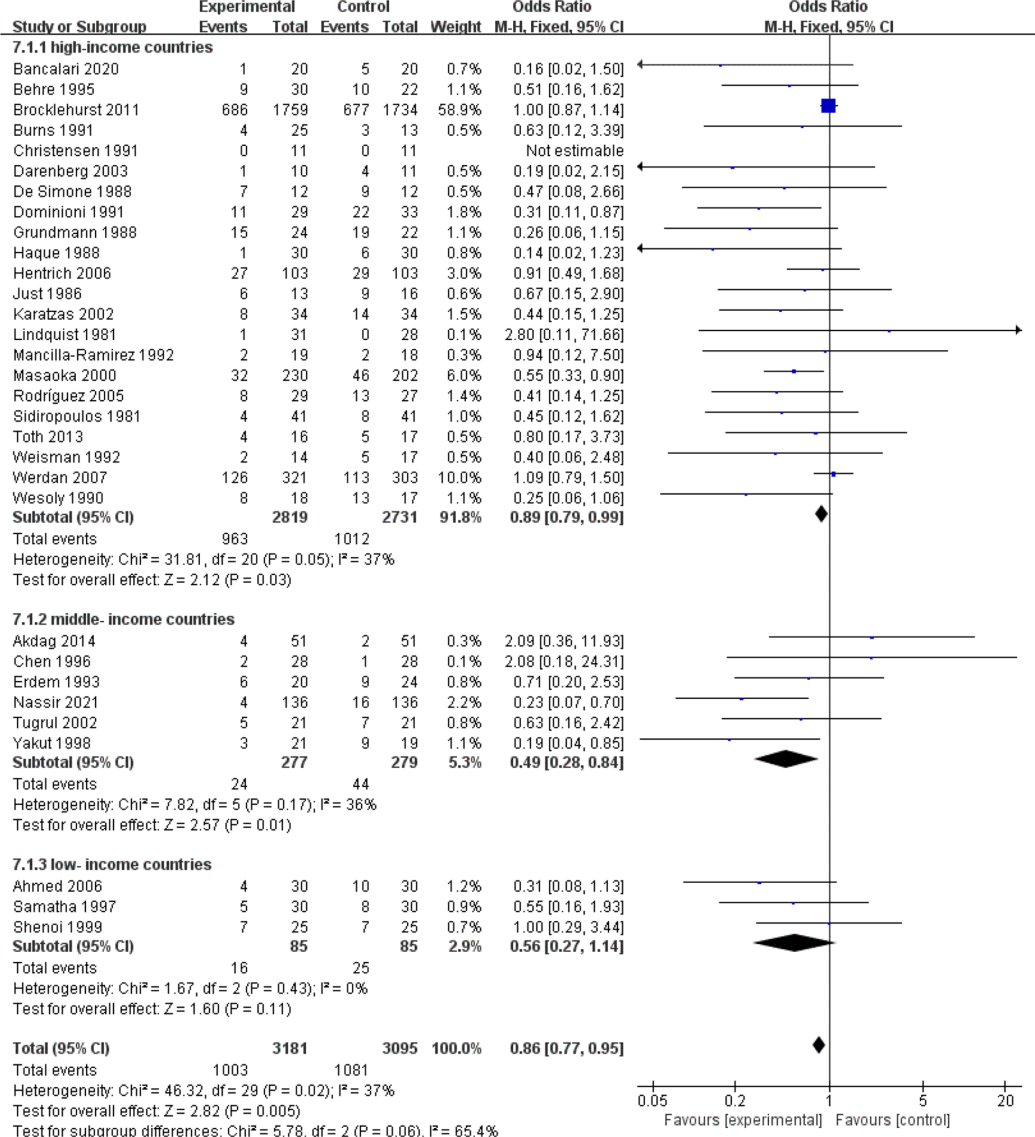
Forest plots of all-cause mortality in 3 different income countries (31 RCTs, n = 6276)

**Fig. 8:**
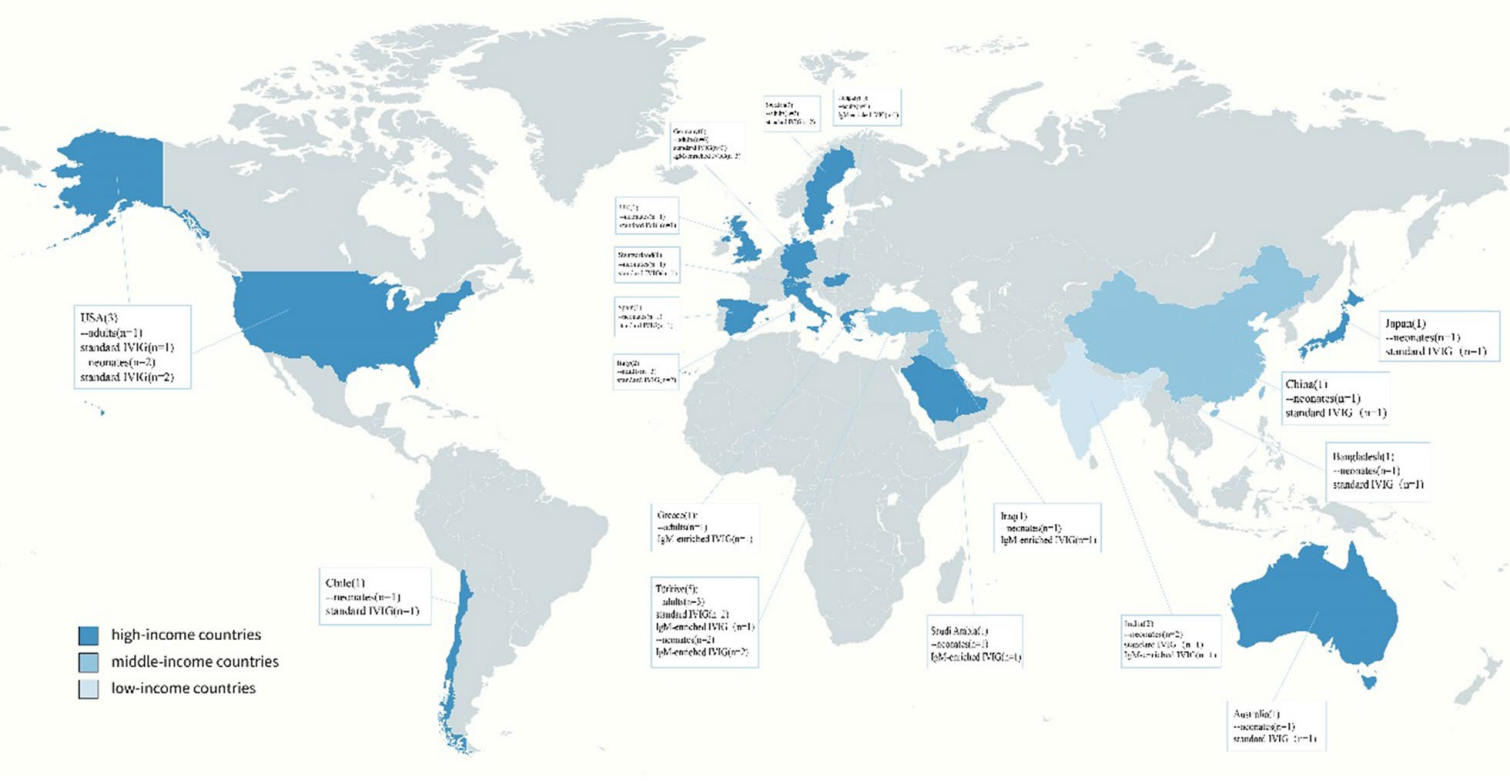
Global RCTs on IVIG for sepsis according to different income levels

Of the 31 RCTs included, 22 were conducted in high-income countries, 6 in middle-income countries and 3 in low-income countries (Figs. 7, 8).The result indicated that IVIG was effective for sepsis in high-income (RR 0.89, 95% CI: 0.79 to 0.99; heterogeneity χ^2^ = 31.81, df = 20, *p* = 0.05, *I*^*2*^ = 37%)and middle-income countries (RR 0.49, 95% CI: 0.28–0.84; heterogeneity χ^2^ = 7.82, df = 5, *p* = 0.17, *I*^*2*^ = 36%), while no benefit was demonstrated in low-income countries (RR 0.56, 95% CI: 0.27–1.14; heterogeneity χ^2^ = 1.67, df = 2, *p* = 0.43, *I* = 0%).

## Discussion

This is a high-quality comprehensive meta-analysis to evaluate the efficacy of IVIG therapy for sepsis. In order to ensure the credibility of the meta-analysis, all nonrandomized controlled trials such as cohort studies were excluded. Similarly, preprints that have not been peer-reviewed were not included.

In this meta-analysis, IVIG treatment significantly reduced all-cause mortality in patients with sepsis compared with placebo plus standard treatment or standard treatment alone. However, in 2016 surviving sepsis campaign suggested against immunoglobulin use in sepsis [16]. The international guidelines followed the principles of the Grading of Recommendations Assessment, Development, and Evaluation system to assess the quality of evidence from high to very low. And the efficacy of IVIG for sepsis showed low quality of evidence because of the significant heterogeneity and inconsistent results. Therefore, large multicentre studies were conducted to further evaluate the efficacy of IVIG in patients with sepsis is essential. The present study was updated to include the latest RCTs and to explore the heterogeneity in terms of different patient ages, IVIG types, and country economic levels to provide more comprehensive evidence on the efficacy of IVIG for sepsis.

When the subjects were subgrouped by 2 different ages, IVIG therapy was associated with a significantly decreased all-cause mortality in adults with sepsis, whereas its efficacy in neonatal sepsis patients was not applicable. From the perspective of disease incidence, neonates are a special group, and they are more likely to develop into sepsis [53]. Studies have demonstrated that the incidence of severe sepsis in children increased from 92.8 cases per 100,000 to 158.7 cases per 100,000 over the past decade [54, 55], whereas the incidence among adults has remained nearly constant over recent decades [56]. In addition, the most significant differences between adults and neonatal sepsis are observed in outcomes [56]. Neonatal sepsis progresses rapidly and has a high short-term mortality rate in terms of mortality, with 40.8% mortality of hospital admission among very preterm infant [57]. However, this high short-term mortality rate is not matched by the fact that the clinical presentation of neonatal sepsis is subtle and may be difficult to identify [56]. Neonates with sepsis could miss the optimal treatment period, so that IVIG is shown to be insufficient in the treatment of sepsis. Furthermore, it is worth noting that compared with adults sepsis, neonatal sepsis is particularly special in the application of plasma derivative due to different primary diseases, pathogenic bacteria and patient condition, in addition to differences in pathogenesis characteristics and clinical manifestations. Providing passive immunity to sick neonates underlies the application of IVIG as a treatment modality for culture-proven or suspected neonatal sepsis in addition to enhancing opsonization, phagocytosis, and complement, promoting antibody-dependent cytotoxicity, modulating T cell and macrophage activity via cytokines, stimulating B cell effector functions, and improving neutrophil-mediated killing [58]. These slow immunomodulatory processes of IVIG may not be applicable in neonatal sepsis with an insidious onset and a very high short-term mortality. Taken together, this may explain the failure of IVIG therapy to reduce all-cause mortality in neonatal sepsis.

When neonatal group was subgrouped by different types of IVIG administration, it was found that standard IVIG did not reduce all-cause mortality in neonatal sepsis in the present study. It was consistent with the results of the meta-analysis by Alejandria [12]. However, for IgM-enriched IVIG, Alejandria concluded that the evidence was still insufficient to support a robust conclusion of benefit. Given that the study by Alejandria included only 3 references on IgM-enriched IVIG for neonatal sepsis, it is necessary to draw reliable conclusions with caution. In the present study, the included references were updated to 5 to show that IgM-enriched IVIG significantly reduced all-cause mortality in neonatal sepsis. Boonsopa [59] concluded that the use of IgM-enriched IVIG reduced the incidence and improved the clinical indicators of sepsis and hypertension in neonates. IgM-enriched IVIG has higher antimicrobial activity and opsonization because of the pentameric structure of IgM to improve activation of the neonatal complement system [18]. These properties confirm the superiority of IgM-enriched IVIG over standard IVIG as a therapy in sepsis.

Notably, accurate estimates of neonatal sepsis burden vary by countries, with different estimates of disease burden reported in high-income countries compared to those reported in low- and middle-income countries [20]. It was found that IVIG was effective for sepsis in high-income and middle-income countries, while no benefit was demonstrated in low-income countries. Rudd [60] observed that morbidity and mortality from sepsis varied considerably across income countries, with the incidence of neonatal sepsis 40 times higher and mortality two times higher in low-income countries than in high-income countries. The disease burden of sepsis may reflect differences in resources and health care settings, other co-infection prevalences, and the spectrum and frequency of comorbidities, which vary from income settings. Although the largest population-based epidemiological data on neonatal sepsis is currently available from a meta-analysis, its estimates of the global burden are considered exploratory due to insufficient data from all low-income countries and most middle-income countries. Therefore, subsequent evaluations of the IVIG efficacy for sepsis need to focus on the impact of the countries income level differences, emphasizing the urgent requirement to obtain data from populations in low- and middle-income countries.

Several limitations may affect the results of our meta-analysis. The collected data were limited because this meta-analysis only included randomised controlled trials with high-quality evidence. Since studies in different subgroups concentrated either in high-income countries or in low- and middle-income countries, the included neonatal RCTs lacked regional representation after subgroup analysis. So the data were only marginally representative. Moreover, we can not exclude the effect of publication bias and the potential effect of some confounders.

## Conclusion

In summary, there is sufficient evidence to support that IVIG reduces sepsis mortality. IgM-enriched IVIG is effective in both adult and neonatal sepsis, while standard IVIG is only effective in adult sepsis. IVIG for sepsis has shown efficacy in high- and middle-income countries, but is still debatable in low-income countries. More RCTs are needed in the future to confirm the true clinical potential of IVIG for sepsis in low-income countries.

### Supplementary Information

**Additional file 1.** The detailed search strategy.

**Additional file 2: Table S1.** Characteristics of RCTs included in this systematic review (adults). **Table S2.** Characteristics of RCTs included in this systematic review (neonates).

## Data Availability

The original data involved in the manuscript can be obtained from references.

## Abbreviations

IVIG: Intravenous immunoglobulins
RCT: Randomized controlled trial
RR: Relative risk
CI: Confidence interval
MD: Mean difference
