# Supplementary material for: Efficacy of IVIG therapy for patients with sepsis: a systematic review and meta-analysis
Source: J Transl Med. 2023 Oct 28;21:765. doi: 10.1186/s12967-023-04592-8 (PMC10612304; doi:10.1186/s12967-023-04592-8)
Supplement: Supplementary file 1 — Additional file 1. The detailed search strategy. [file 12967_2023_4592_MOESM1_ESM.docx]

**The detailed search strategy.**

**Pubmed**

(((((((IVIG[Title/Abstract]) OR (Immunoglobulins, Intravenous[Title/Abstract])) OR (Antibodies, Intravenous[Title/Abstract])) OR (Intravenous Antibodies[Title/Abstract])) OR (Intravenous Immunoglobulin*[Title/Abstract])))) AND ((((Septic Shock[Title/Abstract]) OR (Septicemia[Title/Abstract])) OR (Sepsis[Title/Abstract])))) AND ((((((((((randomized controlled trial[Publication Type]) OR (controlled clinical trial[Publication Type])) OR (randomized[Title/Abstract])) OR (controled[Title/Abstract])) OR (trial[Title/Abstract])) OR (random[Title/Abstract])) OR (placcbo[Title/Abstract])) OR (groups[Title/Abstract])))

Date Janunary, 31, 2023 Results: 169

**Embase**

('sepsis'/exp OR 'septicemia'/exp OR 'septic shock'/exp OR 'pyemia'/exp) AND ('immunoglobulins, intravenous':ti,ab OR ivig:ti,ab OR 'intravenous antibodies':ti,ab OR 'intravenous immunoglobulin':ti,ab OR 'antibodies, intravenous':ti,ab) AND ('randomized controlled trial (topic)'/exp OR placebo:ti,ab OR randomized:ti,ab OR 'controlled clinical trial':ti,ab OR controled:ti,ab OR random:ti,ab OR groups:ti,ab OR trial:ti,ab)

Date Janunary, 31, 2023 Results: 392

**Cochrane**

#1 Immunoglobulins, Intravenous

#2 IVIG

#3 Antibodies, Intravenous

#4 Intravenous Antibodies

#5 Intravenous Immunoglobulin

#6 #1 OR #2 OR #3 OR #4 OR #5

#7 Sepsis

#8 Septic Shock

#9 Septicemia

#10 Pyemia

#11 Pyohemia

#12 #7 OR #8 OR #9 OR #10 OR #11

#13 randomized controlled trial

#14 RCT

#15 controlled clinical trial

#16 placebo

#17 random

#18 groups

#19 #13 OR #14 OR #15 OR #16 OR #17 OR #18

#20 #6 AND #12 AND #19 in Trials

Date Janunary, 31, 2023 Results: 315

**Chinese National Knowledge Infrastructure database**

Immunoglobulins, Intravenous + IVIG + Antibodies, Intravenous + Intravenous Antibodies + Intravenous Immunoglobulin AND Sepsis + Septic Shock + Septicemia + Pyemia + Pyohemia

Date Janunary, 31, 2023 Results: 128
