# Supplementary material for: Efficacy of IVIG therapy for patients with sepsis: a systematic review and meta-analysis
Source: J Transl Med. 2023 Oct 28;21:765. doi: 10.1186/s12967-023-04592-8 (PMC10612304; doi:10.1186/s12967-023-04592-8)
Supplement: Supplementary file 2 — Additional file 2: Table S1. Characteristics of RCTs included in this systematic review (adults). Table S2. Characteristics of RCTs included in this systematic review (neonates). [file 12967_2023_4592_MOESM2_ESM.docx]

**Table S1.** Characteristics of RCTs included in this systematic review(adults)

| **Source** | **Country** | **Study design** | **Total,n** | **Intervention group**  **(1) Number**  **(2) Week-age, mean(SD)**  **(3) IVIG type** | **Control group**  **(1) Number**  **(2) Week-age, mean(SD)**  **(3) Treatment** | **Outcomes** | **Adverse events** |
| --- | --- | --- | --- | --- | --- | --- | --- |
| Hentrich 2006 | Germany | multi-centre RCT | 206 | (1)103  (2)48.8(14.5)  (3) IgM-enriched IVIG: 200 ml initially followed by 11 infusions 100 ml each given every 6 hrs | (1)103  (2)51.0(17.3)  (3)5% albumin | All-cause mortality | allergic event,  allergic reaction,  erythema,  vomiting,et al. |
| Wesoly 1990 | Germany | single-centre RCT | 35 | (1)18  (2)NR  (3) IgM-enriched IVIG | (1)17  (2)NR  (3)placebo | All-cause mortality | NR |
| Toth 2013 | Hungary | single-centre RCT | 33 | (1)16  (2)60(3.25)  (3)IgM-enriched IVIG: 5 ml/kg | (1)17  (2)56(3.75)  (3)placebo | All-cause mortality | NR |
| Rodríguez 2005 | Spain, Argentina | multi-centre RCT | 56 | (1)29  (2)61.3(19.9)  (3) IgM-enriched IVIG:7 ml/kg/day for 5 days | (1)27  (2)65.9(18.2)  (3)5% albumin | All-cause mortality, APACHE II score | NR |

**Table S1.** (continued)

| **Source** | **Country** | **Study design** | **Total,n** | **Intervention group**  **(1) Number**  **(2) Week-age, mean(SD)**  **(3) IVIG type** | **Control group**  **(1) Number**  **(2) Week-age, mean(SD)**  **(3) Treatment** | **Outcomes** | **Adverse events** |
| --- | --- | --- | --- | --- | --- | --- | --- |
| Behre 1995 | Germany | single-centre RCT | 52 | (1)30  (2)NR  (3) IgM-enriched IVIG：total dose of 1.3 litres every 6 hrs for 3 days | (1)22  (2)NR  (3)5% albumin | All-cause mortality | NR |
| Burns 1991 | USA | single-centre RCT | 38 | (1)25  (2)61.5(NR)  (3)standard IVIG:400 mg/kg/day for 3 days | (1)13  (2)59.8(NR)  (3)albumin | All-cause mortality | NR |
| Grundmann 1988 | Germany | single-centre RCT | 46 | (1)24  (2)NR  (3)standard IVIG:0.25 g/kg on day of study entry and the following day | (1)22  (2)NR  (3)albumin | All-cause mortality | NR |
| Just 1986 | German | single-centre RCT | 29 | 1)13  (2)40.2(18.5)  (3)standard IVIG: 100 ml given at 0h, 12h, 24h and 36h | (1)16  (2) 40.2(18.6)  (3) no intervention | All-cause mortality | NR |

**Table S1.** (continued)

| **Source** | **Country** | **Study design** | **Total,n** | **Intervention group**  **(1) Number**  **(2) Week-age, mean(SD)**  **(3) IVIG type** | **Control group**  **(1) Number**  **(2) Week-age, mean(SD)**  **(3) Treatment** | **Outcomes** | **Adverse events** |
| --- | --- | --- | --- | --- | --- | --- | --- |
| Lindquist 1981 | Sweden | single-centre RCT | 59 | (1)31  (2)55.3(3.0)  (3)Standard IVIG:0.15 g/kg infused over 1 hr repeated after 24 and 48 hr | (1)28  (2)57.5(3.3)  (3) antibiotics alone | All-cause mortality | shock,  rigor,  vomiting,  et al. |
| Masaoka 2000 | Japan | multi-centre RCT | 432 | (1)230  (2)NR  (3)standard IVIG：5 g daily for 3 days | (1)202  (2)NR  (3) no treatment | All-cause mortality | nausea  vomiting， pruritus，  dyspnoea |
| De Simone 1988 | Italy | single-centre RCT | 24 | (1)12  (2)45(4)  (3)standard IVIG: 0.4 g/kg on day of admission, 0.2 g/kg after 48 hrs and 0.4 g/kg as needed combined with antibiotics | (1)12  (2)45(5)  (3)antibiotics alone | All-cause mortality | NR |
| Darenberg 2003 | Sweden,  Finland, Norway,  et al. | multi-centre RCT | 21 | (1)10  (2)51.3(11)  (3)standard IVIG:1 g/kg on day 1 and 0.5 g/kg on days 2 and 3 | (1)11  (2)52.6(12)  (3)1% albumin | All-cause mortality | NR |

**Table S1.** (continued)

| **Source** | **Country** | **Study design** | **Total,n** | **Intervention group**  **(1) Number**  **(2) Week-age, mean(SD)**  **(3) IVIG type** | **Control group**  **(1) Number**  **(2) Week-age, mean(SD)**  **(3) Treatment** | **Outcomes** | **Adverse events** |
| --- | --- | --- | --- | --- | --- | --- | --- |
| Dominioni 1991 | Italy | multi-centre RCT | 62 | (1)29  (2)67(10)  (3)standard IVIG: 0.4g/kg on days 0, 1 then 0.2g/kg on day 5 | (1)33  (2)68(12)  (3)albumin in 5% dextrose water | All-cause mortality,  APACHE II score | NR |
| Werdan 2007 | Germany | multi-centre RCT | 624 | (1)321  (2)57.2(13.7)  (3)standard IVIG：600 mg/kg on day 0 and 300 mg/kg on day1 | (1)303  (2)57.7（13.6）  (3) 0.1% serum albumin | All-cause mortality,  APACHE II score | NR |
| Yakut 1998 | Türkiye | ingle-centre RCT | 40 | (1)21  (2)NR  (3)standard IVIG：0.4g/kg on days 0 and 1, 0.2 g/kg on days 2-4 | 1)19  (2)NR  (3)albumin | All-cause mortality | NR |
| Karatzas 2002 | Greece | single-centre RCT | 68 | (1)34(3.33)  (2)50.5(3.33)  (3)IgM-enriched IVIG | (1)34(7.36)  (2)50.7(7.36)  (3)placebo | All-cause mortality, APACHE II score | NR |

**Table S1.** (continued)

| **Source** | **Country** | **Study design** | **Total,n** | **Intervention group**  **(1) Number**  **(2) Week-age, mean(SD)**  **(3) IVIG type** | **Control group**  **(1) Number**  **(2) Week-age, mean(SD)**  **(3) Treatment** | **Outcomes** | **Adverse events** |
| --- | --- | --- | --- | --- | --- | --- | --- |
| Tugrul 2002 | Türkiye | single-centre RCT | 42 | (1)21  (2)42(18)  (3) IgM-enriched IVIG:5 ml/kg/day infused over 6 hrs and repeated for 3 consecutive days | (1)21  (2)49.3(20.6)  (3)standard sepsis therapy | All-cause mortality,  APACHE II score | hypotension and hypoglycaemia |

Abbreviation: NR, Not reported.

**Table S2.** Characteristics of RCTs included in this systematic review(neonates)

| **Source** | **Country** | **Study design** | **Total,n** | **Intervention group**  **(1) Number**  **(2) Week-age, mean(SD)**  **(3) IVIG type** | **Control group**  **(1) Number**  **(2) Week-age, mean(SD)**  **(3) Treatment** | **Outcomes** | **Adverse events** |
| --- | --- | --- | --- | --- | --- | --- | --- |
| Weisman 1992 | USA | multi-centre RCT | 31 | (1)14  (2)28.5(2.8)  (3)Standard IVIG:500mg/kg single infusion for 2 hrs | (1)17  (2)28.2(2.6)  (3)Albumin | All-cause mortality,  length of hospital stay | infusion-related adverse reaction:  hypotension,  hypoglycaemia |
| Bancalari 2020 | Chile | single-centre RCT | 40 | (1)20  (2)29.8(NR)  (3)Standard IVIG:combined with antimicrobials | (1)20  (2)29.6(NR)  (3)Antibiotic treatment | All-cause mortality | NR |
| Shenoi 1999 | India | multi-centre RCT | 50 | (1)25  (2)NR  (3)Standard IVIG:1g/kg for 3 consecutive days | (1)25  (2)NR  (3)Placebo using 0.15% saline in 10% dextrose | All-cause mortality | NR |
| Brocklehurst 2011 | UK,  Australia,  Argentina  et al. | multi-centre RCT | 3493 | (1)1759  (2)1.21(NR)  (3)Standard IVIG:500 mg/kg for over 4 to 6 hrs, repeated 48 hrs later | (1)1734  (2)1.21(NR)  (3)0.2% albumin solution in normal saline | All-cause mortality | NR |

**Table S2.** (continued)

| **Source** | **Country** | **Study design** | **Total,n** | **Intervention group**  **(1) Number**  **(2) Week-age, mean(SD)**  **(3) IVIG type** | **Control group**  **(1) Number**  **(2) Week-age, mean(SD)**  **(3) Treatment** | **Outcomes** | **Adverse events** |
| --- | --- | --- | --- | --- | --- | --- | --- |
| Mancilla-Ramirez 1992 | Spain | single-centre RCT | 37 | (1)19  (2)NR  (3)Standard IVIG：500 mg/kg single dose | (1)18  (2)NR  (3)10% maltose | All-cause mortality,  length of hospital stay | NR |
| Chen 1996 | China | single-centre RCT | 56 | (1)28  (2)NR  (3)Standard IVIG | (1)28  (2)NR  (3)Placebo | All-cause mortality,  length of hospital stay | NR |
| Ahmed 2006 | Bangladesh | single-centre RCT | 60 | (1)30  (2)1.45(0.29)  (3)Standard IVIG：500 mg/kg once daily for 3 consecutive days | (1)30  (2)1.56(0.30)  (3)Placebo | All-cause mortality,  length of hospital stay | NR |
| Christensen 1991 | USA | single-centre RCT | 22 | (1)11  (2)0.17(0.06)  (3)Standard IVIG: a single dose of 750 mg/kg | (1)11  (2)0.25(0.16)  (3)0.1% albumin in a sterile 10% maltose solution | All-cause mortality | NR |

**Table S2.** (continued)

| **Source** | **Country** | **Study design** | **Total,n** | **Intervention group**  **(1) Number**  **(2) Week-age, mean(SD)**  **(3) IVIG type** | **Control group**  **(1) Number**  **(2) Week-age, mean(SD)**  **(3) Treatment** | **Outcomes** | **Adverse events** |
| --- | --- | --- | --- | --- | --- | --- | --- |
| Sidiropoulos 1981 | Switzerland | single-centre RCT | 82 | (1)41  (2)NR  (3)Standard IVIG：preterm infants re ceived 0.5 g/day for 6 days and term infants 1.0 g/day for 6 days | (1)41  (2)NR  (3)Placebo | All-cause mortality | NR |
| Haque 1988 | Saudi Arabia | single-centre RCT | 60 | (1)30  (2)33.4(NR)  (3)IgM-enriched  immunoglobulin:500 mg/kg single infusion for 2 hrs | (1)30  (2)35(NR)  (3)10% dextrose placebo | All-cause mortality | NR |
| Erdem 1993 | Türkiye | single-centre RCT | 44 | (1)202  (2)NR  (3)IgM-enriched  immunoglobulin  (Pentaglobin):5ml/kg/d for 3 days | (1)24  (2)NR  (3)no intervention | All-cause  mortality | NR |

**Table S2.** (continued)

| **Source** | **Country** | **Study design** | **Total,n** | **Intervention group**  **(1) Number**  **(2) Week-age, mean(SD)**  **(3) IVIG type** | **Control group**  **(1) Number**  **(2) Week-age, mean(SD)**  **(3) Treatment** | **Outcomes** | **Adverse events** |
| --- | --- | --- | --- | --- | --- | --- | --- |
| Nassir 2021 | Iraq | multi-centre RCT | 272 | (1)136  (2)1.86(0.61)  (3)IgM-enriched immunoglobulin | (1)136  (2)1.73(0.50)  (3) Placebo | All-cause mortality | NR |
| Akdag 2014 | Türkiye | single-centre RCT | 102 | (1)51  (2)30(NR)  (3)IgM-enriched immunoglobulin:250 mg/kg over 4 hours, daily for three consecutive day | (1)51  (2)31(NR)  (3)Placebo | All-cause mortality | NR |
| Samatha 1997 | India | single-centre RCT | 60 | (1)30  (2)NR  (3)IgM-enriched immunoglobulin(Pentaglobin) 5 ml/kg/d as single dose infused at 1.7 ml/kg/hr for 3 con secutive days | (1)30  (2)NR  (3)Antibiotics and supportive treatment | All-cause mortality,  length of hospital stay | NR |

Abbreviation: NR, Not reported.
